# Impact of Feeding Strategies With Acid Suppression on Esophageal Reflexes in Human Neonates With Gastroesophageal Reflux Disease: A Single-Blinded Randomized Clinical Trial

**DOI:** 10.14309/ctg.0000000000000249

**Published:** 2020-11-05

**Authors:** Sudarshan R. Jadcherla, Kathryn A. Hasenstab, Ish K. Gulati, Roseanna Helmick, Haluk Ipek, Vedat Yildiz, Lai Wei

**Affiliations:** 1 Innovative Infant Feeding Disorders Research Program, Nationwide Children's Hospital, Columbus, Ohio, USA;; 2 Center for Perinatal Research, The Research Institute at Nationwide Children's Hospital, Columbus, Ohio, USA;; 3 Division of Neonatology, Nationwide Children's Hospital Columbus, Ohio, USA;; 4 Division of Pediatric Gastroenterology, Hepatology, and Nutrition, Department of Pediatrics, The Ohio State University College of Medicine, Columbus, Ohio, USA;; 5 Biostatistics Resource at Nationwide Children's Hospital (BRANCH), Columbus, Ohio, USA;; 6 Center for Biostatistics, Department of Biomedical Informatics, The Ohio State University College of Medicine, Columbus, Ohio, USA.

## Abstract

**INTRODUCTION:**

Aims were to test hypothesis that esophageal provocation-induced reflexes are superior with acid suppression plus feeding modifications vs acid suppression alone among infants treated for gastroesophageal reflux disease (GERD).

**METHODS:**

Infants (N = 49, 41.3 ± 2.6 of postmenstrual age) with acid reflux index >3% underwent longitudinal motility testing (weeks 0 and 5) with graded midesophageal provocation to test randomly allocated therapies (4 weeks' proton pump inhibitor [PPI] ± feeding modifications) on sensory-motor aerodigestive reflexes. Feeding modification included restricted fluid volume <140 mL/kg per day, fed over 30 minutes in right lateral position and supine postprandial position. Primary motility outcome was frequency-occurrence of peristaltic reflex. Secondary outcomes included upper esophageal sphincter contractile reflex, lower esophageal sphincter (LES) relaxation reflex, respiratory change, and symptom characteristics.

**RESULTS:**

Treatment groups did not differ for primary outcome (odds ratio = 0.8, 95% confidence interval 0.4–1.6, *P* = 0.99) or secondary outcomes (all *P* > 0.05). For both treatment groups at follow-up, distal esophageal contraction and LES tone decreased, and LES relaxation reflex occurrence is less frequent (all *P* < 0.05). In a subgroup analysis, comparing infants with PPI washout (N = 40) vs with continued (N = 9) PPI therapy, no differences were noted for aerodigestive reflex response frequency-occurrence (all *P* > 0.05).

**DISCUSSION:**

In infants with GERD, feeding modification with acid suppression is not superior to acid suppression alone in modifying aerodigestive reflexes (frequency, sensation, or magnitude). Contiguous areas targeted by GER, i.e., LES and distal esophageal functions, worsened at follow-up for both groups despite PPI therapy. Maturation is likely the key factor for GERD resolution in infants, justifying the use of placebo in clinical trials for objectively determined GERD.

## INTRODUCTION

Diagnosis and management of gastroesophageal reflux disease (GERD) in infants in the neonatal intensive care unit is challenging. Diagnosis is often empiric and symptom based, resulting in widely variable postdischarge management strategies that may include combinations of conservative, pharmacologic, or surgical therapies (1–9). In infants, conservative management strategies are often used first and include feeding modifications (FMs) such as restricted total fluid volume intake, prolonged feeding duration, and altered body position during and after feeds (1,10–12). Prolonged dietary and positional approaches may be ineffective, have nutritional and growth consequences, escalate burden, and impact discharge planning and parent teaching (10,13–19). If conservative management strategies fail, pharmacologic therapy or acid suppressive medication for 4–8 weeks is considered and often prescribed off-label (1,12,20). Although the adverse unintended consequences of acid suppression are known, the true indications and treatment duration remain unknown (21). Collectively, all these non–evidence-based approaches escalate the economic burden, and the label of GERD diagnosis increases hospitalization by a month costing ∼70K US dollars more per infant (1,12,22,23).

GERD can be objectively determined by pH-impedance testing using the acid reflux index (ARI) as a measure of the percent of time the esophagus is exposed to acid GER (20,24). The North American Society for Pediatric Gastroenterology, Hepatology, and Nutrition has previously proposed values as guidelines rather than absolutes using ARI <3% as normal, ARI between 3% and 7% as indeterminate, and ARI >7% as abnormal for pediatric and infant populations (24). Common GER mechanisms in infants are transient lower esophageal sphincter (LES) relaxation or hypotonic LES (25,26). In addition, frequent esophageal exposure to acid may cause inflammation (27) and decreased function locally and in extraesophageal and supraesophageal areas.

When GER events occur, numerous reflexes of the respiratory and digestive systems called aerodigestive reflexes may be activated including upper esophageal sphincter contractile reflex (UESCR), peristaltic reflex, LES relaxation reflex (LESRR), glottal closure manifesting as apnea, and symptoms such as cough or sneeze (28–35). Although it is known aerodigestive reflexes improve with maturation in preterm infants (28–35), it is unknown how GERD therapies modify these reflexes.

Our aims were to evaluate and compare esophageal provocation–induced aerodigestive reflexes in infants treated with acid suppression alone (conventional approach) vs acid suppression with an FM bundle (study approach). Our a priori hypothesis of this prospective study was that midesophageal stimulation–induced aerodigestive reflexes are superior in the study approach vs the conventional approach. The rationale for the study hypothesis was to interrogate whether either GERD therapy (pharmacologic or pharmacologic plus conservative management) modified sensitivity or motor characteristics of pharyngoesophageal motility which is central to aerodigestive safety; if so, then, designing specific therapies will be possible; if not, then, a case can be made for placebo-controlled randomized clinical trials in infants with objective criteria for true GERD.

## METHODS

### Participants and setting

Studies were part of a single-center, single-blinded, and randomized clinical trial (clinicaltrials.gov, NCT02486263) in convalescing neonatal intensive care unit infants (including term and preterm born infants) with GERD to test the effectiveness of acid suppressive therapy with a FM bundle. Clinical outcomes of this trial have been previously reported (36), while the current report focuses on the mechanistic motility outcomes. Inclusion criteria for this report for hospitalized infants are as follows: (i) To accommodate for gestational and postnatal maturation, 2 age-related metrics were used for trial inclusion criteria—gestational age (GA) ≤42.0 weeks and postmenstrual age (PMA) between 34 and 60 weeks. GA is birth age and measured as the duration from conception to birth. PMA accounts for gestational and postnatal maturation and is used as a common age term at time of evaluation for preterm (<37.0 weeks' GA) and full-term (37.0–42.0 weeks' GA) born infants. In addition, maturational functions of vagovagal reflexes are defined by PMA; (ii) Clinical symptoms of GERD with physician intention-to-treat and pH-impedance testing confirmed ARI ≥3% (24,37–39); (iii) at time of evaluation receiving feeding intake volume of full enteral feeds ≥150 mL/kg per day, on room air or supplemental oxygen up to 1 L per minute; and (iv) had longitudinal motility testing (at week 0—baseline and week 5—follow-up). Exclusion criteria were infants with (i) known genetic, metabolic, or syndromic diseases; (ii) neurological diseases including ≥grade III intraventricular hemorrhage or perinatal asphyxia; and (iii) gastrointestinal (GI) malformations or surgical GI conditions.

Studies were performed at the Innovative Infant Feeding Disorders Research Program at Nationwide Children's Hospital, Columbus, OH. Institutional review board approval was obtained (IRB# 11-00734). Study recruitment and criteria adherence were reported to the Data Safety Monitoring Board through face-to-face meetings and paper reports on a quarterly basis. Written, signed, informed parental consent was obtained before the study. Health Insurance Portability and Accountability Act guidelines were followed.

### Study design

**Figure 1.**
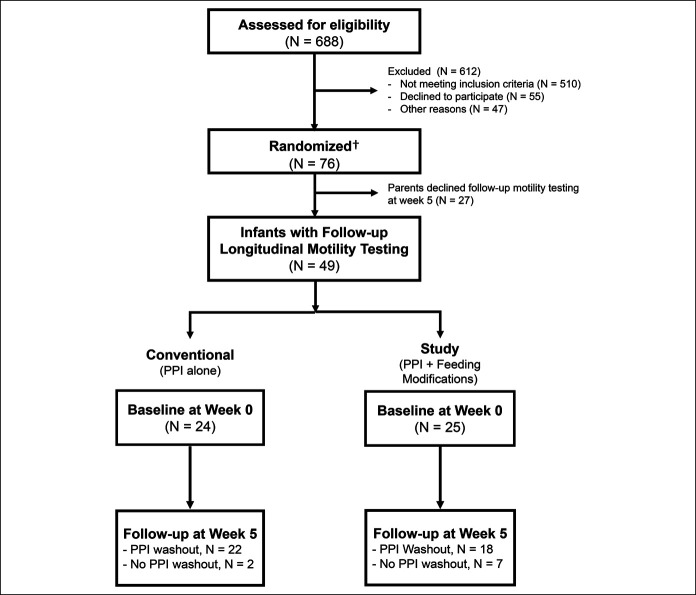
Study flow diagram. †Clinical outcomes (feeding status and symptom scores) of this randomized controlled trial have been previously reported (36), while the focus of this current report is the motility outcomes of those infants who underwent longitudinal motility testing to determine the effect of conventional (proton pump inhibitor [PPI] alone) and study treatments (PPI + feeding modifications). Note of the 49 infants studied at week 5: 40 infants had 1 week of PPI washout as intended and were analyzed for all motility outcomes. Subanalysis was performed for the remaining 9 infants who did not receive washout due to parental refusal to discontinue medication.

Only those infants who have completed longitudinal motility testing were evaluated for motility outcomes (Figure 1). Longitudinal motility testing (before therapy at week 0 and after therapy at week 5) is included in this report. Demographic and clinical data were managed using research electronic data capture tools (40). Motility data were managed by MMS analysis software (v. 2.04; Laborie Medical Technologies, Mississauga, ON, Canada).

#### Intervention protocol.

Infants were randomized to conventional (acid suppression alone) or study (acid suppression + FMs) using a computer generated allocation ratio (1:1) stratified by ARI severity (indeterminate acid reflux: 3%–7% or severe acid reflux >7%) and birth gestation (preterm or full-term born) to ensure equal distribution. Infants were treated for a duration of 4 weeks. Both groups received acid suppressive therapy (omeprazole, 0.75 mg/kg per dose b.i.d. off label), while the study group also received an FM bundle which included restricted feeding volume intake to ≤140 mL/kg per day (7), feeding in right lateral position to facilitate intraprandial gastric emptying (41), over a feeding duration of at least 30 minutes, and supine postprandial position (7).

#### Pharyngoesophageal motility study protocol.

Subjects underwent longitudinal provocative pharyngoesophageal motility testing at week 0 (inception) and week 5 (4 weeks of therapy + 1-week proton pump inhibitor [PPI] washout) (Figure 1). As validated, a custom-designed silicone catheter (Dentsleeve International, Mui Scientific, ON, Canada) with 5 pressure sensors (pharynx; proximal, middle, and distal esophagus; and stomach), sleeves (UES and LES), and midesophageal-infusion port was connected to a water perfusion motility system (Solar GI; Laborie Medical Technologies) for intraluminal pressure recordings and analysis of pharyngeal, esophageal, and sphincter responses (28–35). The catheter was positioned by the study physician through the nasogastric route using the pull-through technique (42). Respiratory inductance plethysmography (Respitrace, Viasys, Conshohocken, PA) and nasal thermistor (Integra Life Sciences, Plainsboro, NJ) recorded respiratory rhythm changes (12,31,43–46). Midesophageal-infusion protocol included graded volumes of air (0.1, 0.5, 1.0, 2.0, and 5.0 mL), water (0.1, 0.5, 1.0, and 2.0 mL), and apple juice (0.1, 0.5, 1.0, and 2.0 mL) in triplicate (28–35).

### Data analysis and rigor

Study staff performing data analysis were blinded to study allocation. Motility outcomes measured upon midesophageal stimulation included metrics for presence of peristaltic reflex, UES function, esophageal body function, LES function, respiratory rhythm changes, and symptoms (28–35,47–50). Briefly, (i) response occurrence (%) was calculated for esophago-deglutition reflex (EDR) (28,29,32,47), secondary peristalsis (SP) (28,32,47,48), UESCR (12,28,34,48,50), esophageal body polymorphic waveform (51), LESRR (12,33,52), respiratory response (44–46,53), and symptoms (e.g., crying, movement, cough, arching, and irritability), (ii) sensory metrics included threshold volume (in milliliters) defined as the minimum volume resulting in a response (28,30,48), and response latency (in seconds) defined as the time between infusion-onset and response-onset (12,43–46,54), and (iii) response magnitude (duration and pressure) for UESCR, esophageal body (proximal, middle, and distal esophagus), LESRR, and respiratory responses. Response duration (in seconds) was defined as time between response-onset and response cessation (45,46,52,54), and response pressure change (in mmHg) was defined as maximum (contraction) or minimum (relaxation) pressure (12,32,34,52,55). Primary motility outcome was frequency-occurrence of peristaltic response (EDR or SP).

### Statistical analysis

Descriptive statistics are reported as median (range [min–max]), mean ± SD or %. Least square means ± SE are reported for continuous sensory and response magnitude data. Odds ratios with 95% confidence interval are reported for categorical response frequency data. For the group comparisons of patient characteristics, two-sample *t* tests were used for the continuous variables and χ^2^ or Fisher exact tests were used for the categorical variables. For the within-group comparisons of demographic data, the paired *t* test and Generalized Estimation Equation approach were used for the comparison of differences within (week 5 vs week 0) and between (change from baseline at week 0 to follow-up at week 5) intervention groups for response frequency-occurrence outcomes, so as to predict the likelihood of the specific response. Repeated measures ANOVA was used for the comparison of differences within (week 5 vs week 0) and between (change from baseline at week 0 to follow-up at week 5) intervention groups for sensory metric motility outcomes and motility response magnitude variables. Effect of PPI on response frequency-occurrence was also examined using Generalized Estimation Equation approach. Compound symmetry is specified for the covariance structure of the repeated data. Bonferroni correction was used to adjust *P* values to conserve the overall type I error at 0.05. Adjusted *P* values <0.05 were considered statistically significant. Statistical Analysis Software, version 9.4 (SAS Institute, Cary, NC) was used.

## RESULTS

### Participant characteristics

**Figure 2.**
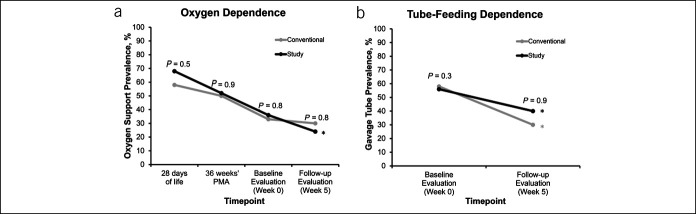
Impact of interventions (conventional or study) on airway and digestive support systems using supplemental oxygen and tube feeding, respectively. Conventional treatment: proton pump inhibitor (PPI) alone, study treatment: PPI + feeding modification bundle. *Denotes *P* < 0.05 over time. Note in (**a**) the need for supplemental oxygen decreases over time in conventional (*P* = 0.14) and in study (*P* = 0.03) group, and in (**b**) the need for tube feeding decreases over time in conventional (*P* = 0.01) and in study (*P* = 0.03). Maturation modifies the changes in airway and digestive needs and not the feeding modifications.

Analysis was performed for a total of 98 motility studies from 49 infants who have completed longitudinal motility testing and is included in this report (Figure 1). Demographic and clinical outcome characteristics did not significantly differ between conventional (PPI alone) and study (PPI + FMs) arms at birth, week 0, week 5, or discharge (Table 1). PPI dosage (mg/kg per dose b.i.d.) for the conventional group was 0.75 (0.75–1.0) vs 0.75 (0.75–1.5) for the study group, *P* = 0.44. Comparison of oxygen and tube-feeding dependence within and between the groups is shown (Figure 2).

**Table 1.**
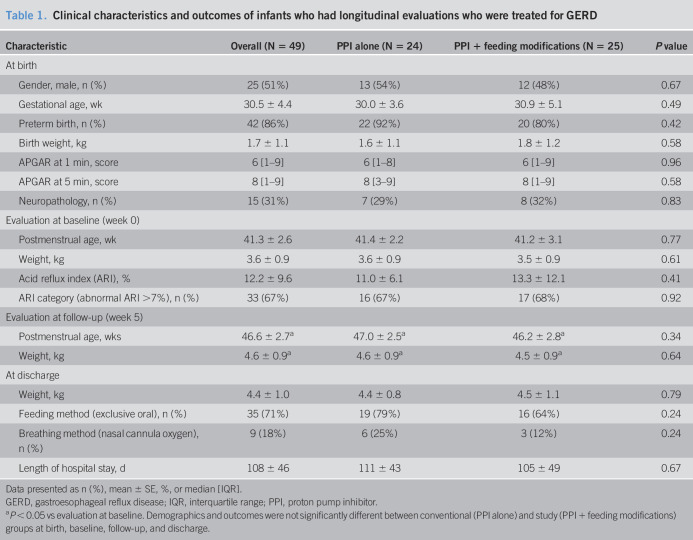
Clinical characteristics and outcomes of infants who had longitudinal evaluations who were treated for GERD

Characteristic	Overall (N = 49)	PPI alone (N = 24)	PPI + feeding modifications (N = 25)	*P* value
At birth				
Gender, male, n (%)	25 (51%)	13 (54%)	12 (48%)	0.67
Gestational age, wk	30.5 ± 4.4	30.0 ± 3.6	30.9 ± 5.1	0.49
Preterm birth, n (%)	42 (86%)	22 (92%)	20 (80%)	0.42
Birth weight, kg	1.7 ± 1.1	1.6 ± 1.1	1.8 ± 1.2	0.58
APGAR at 1 min, score	6 [1–9]	6 [1–8]	6 [1–9]	0.96
APGAR at 5 min, score	8 [1–9]	8 [3–9]	8 [1–9]	0.58
Neuropathology, n (%)	15 (31%)	7 (29%)	8 (32%)	0.83
Evaluation at baseline (week 0)				
Postmenstrual age, wk	41.3 ± 2.6	41.4 ± 2.2	41.2 ± 3.1	0.77
Weight, kg	3.6 ± 0.9	3.6 ± 0.9	3.5 ± 0.9	0.61
Acid reflux index (ARI), %	12.2 ± 9.6	11.0 ± 6.1	13.3 ± 12.1	0.41
ARI category (abnormal ARI >7%), n (%)	33 (67%)	16 (67%)	17 (68%)	0.92
Evaluation at follow-up (week 5)				
Postmenstrual age, wks	46.6 ± 2.7^a^	47.0 ± 2.5^a^	46.2 ± 2.8^a^	0.34
Weight, kg	4.6 ± 0.9^a^	4.6 ± 0.9^a^	4.5 ± 0.9^a^	0.64
At discharge				
Weight, kg	4.4 ± 1.0	4.4 ± 0.8	4.5 ± 1.1	0.79
Feeding method (exclusive oral), n (%)	35 (71%)	19 (79%)	16 (64%)	0.24
Breathing method (nasal cannula oxygen), n (%)	9 (18%)	6 (25%)	3 (12%)	0.24
Length of hospital stay, d	108 ± 46	111 ± 43	105 ± 49	0.67

Data presented as n (%), mean ± SE, %, or median [IQR].

GERD, gastroesophageal reflux disease; IQR, interquartile range; PPI, proton pump inhibitor.

a *P* < 0.05 vs evaluation at baseline. Demographics and outcomes were not significantly different between conventional (PPI alone) and study (PPI + feeding modifications) groups at birth, baseline, follow-up, and discharge.

**Figure 3.**
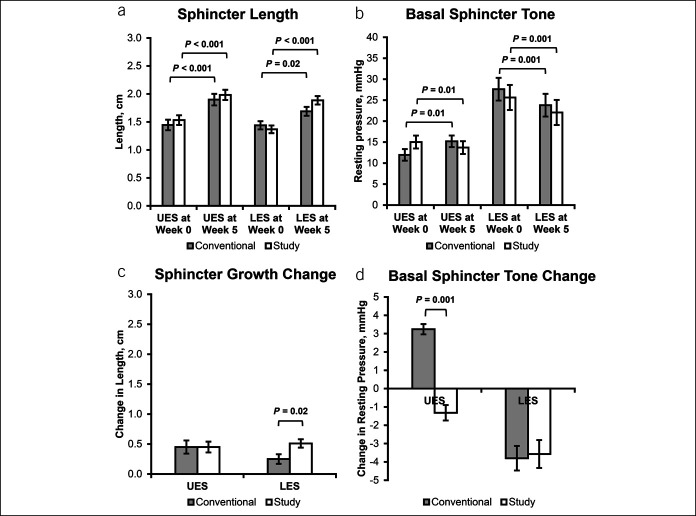
Physiological characteristics of esophageal sphincters. Conventional treatment: PPI alone, study treatment: PPI + feeding modification bundle. For (**a**) and (**b**), comparisons were performed within and between groups; (**a**) UES and LES sphincter length increased with maturation in infants treated for GERD regardless of intervention type. (**b**) Differences were noted with UES basal tone across maturation, regardless of intervention. Also, note LES tone decreased across maturation in infants treated for GERD in both treatment groups. For (**c**) and (**d**), change was calculated as the difference between week 5 and week 0 for both intervention groups. (**c**) LES growth was greater in the study group. (**d**) UES tone increased in the conventional group and decreased in the study group, and LES tone decreased for both groups. GERD, gastroesophageal reflux disease; LES, lower esophageal sphincter; PPI, proton pump inhibitor; UES, upper esophageal sphincter.

Sphincter growth and basal tone of the esophageal sphincters are shown (Figure 3). Note that sphincter lengths increased at follow-up for both interventions, while LES tone decreased at follow-up for both interventions.

### Effect of therapies and maturation on motility outcomes

Of the 49 infants studied, 40 (81.6%) infants received 4 weeks of PPI therapy + 1 week of washout and analyzed for motility outcomes in Tables 2–7. Separate analysis of response frequency-occurrence was performed (Table 8) for the remaining 9 (18.4%) infants who remained on PPI therapy at week 5 due to parental refusal to discontinue PPI therapy.

**Table 2.**
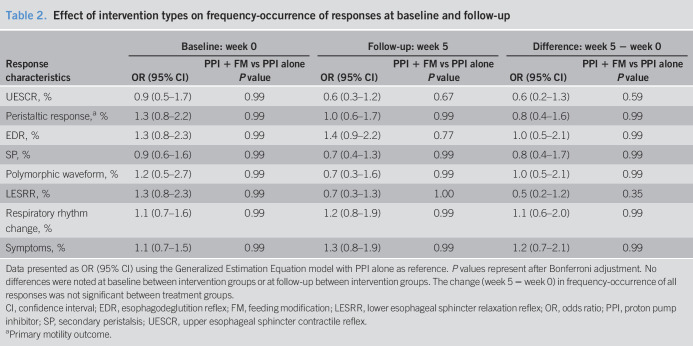
Effect of intervention types on frequency-occurrence of responses at baseline and follow-up

Response characteristics	Baseline: week 0		Follow-up: week 5		Difference: week 5 − week 0	
	OR (95% CI)	PPI + FM vs PPI alone *P* value	OR (95% CI)	PPI + FM vs PPI alone *P* value	OR (95% CI)	PPI + FM vs PPI alone *P* value
UESCR, %	0.9 (0.5–1.7)	0.99	0.6 (0.3–1.2)	0.67	0.6 (0.2–1.3)	0.59
Peristaltic response,^a^ %	1.3 (0.8–2.2)	0.99	1.0 (0.6–1.7)	0.99	0.8 (0.4–1.6)	0.99
EDR, %	1.3 (0.8–2.3)	0.99	1.4 (0.9–2.2)	0.77	1.0 (0.5–2.1)	0.99
SP, %	0.9 (0.6–1.6)	0.99	0.7 (0.4–1.3)	0.99	0.8 (0.4–1.7)	0.99
Polymorphic waveform, %	1.2 (0.5–2.7)	0.99	0.7 (0.3–1.6)	0.99	1.0 (0.5–2.1)	0.99
LESRR, %	1.3 (0.8–2.3)	0.99	0.7 (0.3–1.3)	1.00	0.5 (0.2–1.2)	0.35
Respiratory rhythm change, %	1.1 (0.7–1.6)	0.99	1.2 (0.8–1.9)	0.99	1.1 (0.6–2.0)	0.99
Symptoms, %	1.1 (0.7–1.5)	0.99	1.3 (0.8–1.9)	0.99	1.2 (0.7–2.1)	0.99

Data presented as OR (95% CI) using the Generalized Estimation Equation model with PPI alone as reference. *P* values represent after Bonferroni adjustment. No differences were noted at baseline between intervention groups or at follow-up between intervention groups. The change (week 5 − week 0) in frequency-occurrence of all responses was not significant between treatment groups.

CI, confidence interval; EDR, esophagodeglutition reflex; FM, feeding modification; LESRR, lower esophageal sphincter relaxation reflex; OR, odds ratio; PPI, proton pump inhibitor; SP, secondary peristalsis; UESCR, upper esophageal sphincter contractile reflex.

a Primary motility outcome.

**Table 3.**
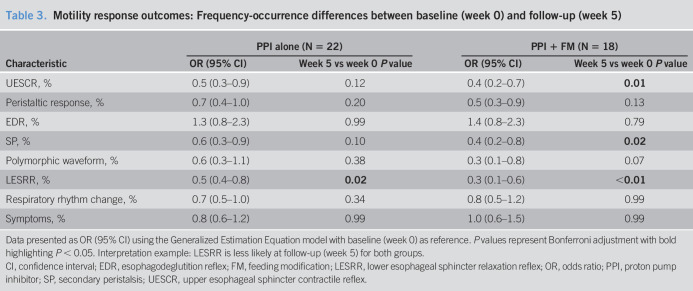
Motility response outcomes: Frequency-occurrence differences between baseline (week 0) and follow-up (week 5)

Characteristic	PPI alone (N = 22)		PPI + FM (N = 18)	
	OR (95% CI)	Week 5 vs week 0 *P* value	OR (95% CI)	Week 5 vs week 0 *P* value
UESCR, %	0.5 (0.3–0.9)	0.12	0.4 (0.2–0.7)	**0.01**
Peristaltic response, %	0.7 (0.4–1.0)	0.20	0.5 (0.3–0.9)	0.13
EDR, %	1.3 (0.8–2.3)	0.99	1.4 (0.8–2.3)	0.79
SP, %	0.6 (0.3–0.9)	0.10	0.4 (0.2–0.8)	**0.02**
Polymorphic waveform, %	0.6 (0.3–1.1)	0.38	0.3 (0.1–0.8)	0.07
LESRR, %	0.5 (0.4–0.8)	**0.02**	0.3 (0.1–0.6)	**<0.01**
Respiratory rhythm change, %	0.7 (0.5–1.0)	0.34	0.8 (0.5–1.2)	0.99
Symptoms, %	0.8 (0.6–1.2)	0.99	1.0 (0.6–1.5)	0.99

Data presented as OR (95% CI) using the Generalized Estimation Equation model with baseline (week 0) as reference. *P* values represent Bonferroni adjustment with bold highlighting *P* < 0.05. Interpretation example: LESRR is less likely at follow-up (week 5) for both groups.

CI, confidence interval; EDR, esophagodeglutition reflex; FM, feeding modification; LESRR, lower esophageal sphincter relaxation reflex; OR, odds ratio; PPI, proton pump inhibitor; SP, secondary peristalsis; UESCR, upper esophageal sphincter contractile reflex.

**Table 4.**
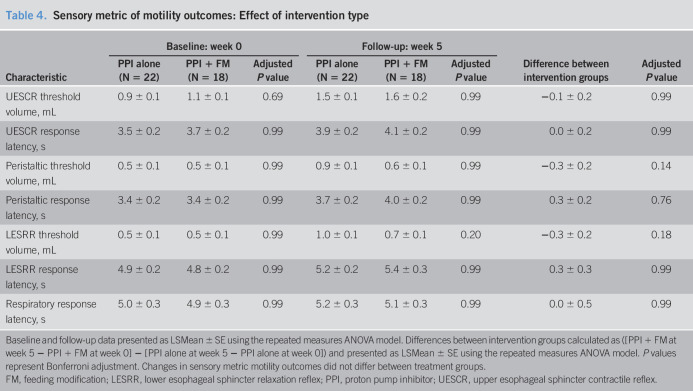
Sensory metric of motility outcomes: Effect of intervention type

Characteristic	Baseline: week 0			Follow-up: week 5			Difference between intervention groups	Adjusted *P* value
	PPI alone (N = 22)	PPI + FM (N = 18)	Adjusted *P* value	PPI alone (N = 22)	PPI + FM (N = 18)	Adjusted *P* value		
UESCR threshold volume, mL	0.9 ± 0.1	1.1 ± 0.1	0.69	1.5 ± 0.1	1.6 ± 0.2	0.99	−0.1 ± 0.2	0.99
UESCR response latency, s	3.5 ± 0.2	3.7 ± 0.2	0.99	3.9 ± 0.2	4.1 ± 0.2	0.99	0.0 ± 0.2	0.99
Peristaltic threshold volume, mL	0.5 ± 0.1	0.5 ± 0.1	0.99	0.9 ± 0.1	0.6 ± 0.1	0.99	−0.3 ± 0.2	0.14
Peristaltic response latency, s	3.4 ± 0.2	3.4 ± 0.2	0.99	3.7 ± 0.2	4.0 ± 0.2	0.99	0.3 ± 0.2	0.76
LESRR threshold volume, mL	0.5 ± 0.1	0.5 ± 0.1	0.99	1.0 ± 0.1	0.7 ± 0.1	0.20	−0.3 ± 0.2	0.18
LESRR response latency, s	4.9 ± 0.2	4.8 ± 0.2	0.99	5.2 ± 0.2	5.4 ± 0.3	0.99	0.3 ± 0.3	0.99
Respiratory response latency, s	5.0 ± 0.3	4.9 ± 0.3	0.99	5.2 ± 0.3	5.1 ± 0.3	0.99	0.0 ± 0.5	0.99

Baseline and follow-up data presented as LSMean ± SE using the repeated measures ANOVA model. Differences between intervention groups calculated as ([PPI + FM at week 5 − PPI + FM at week 0] − [PPI alone at week 5 − PPI alone at week 0]) and presented as LSMean ± SE using the repeated measures ANOVA model. *P* values represent Bonferroni adjustment. Changes in sensory metric motility outcomes did not differ between treatment groups.

FM, feeding modification; LESRR, lower esophageal sphincter relaxation reflex; PPI, proton pump inhibitor; UESCR, upper esophageal sphincter contractile reflex.

**Table 5.**
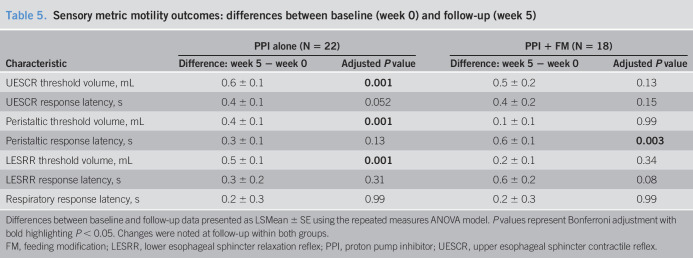
Sensory metric motility outcomes: differences between baseline (week 0) and follow-up (week 5)

Characteristic	PPI alone (N = 22)		PPI + FM (N = 18)	
	Difference: week 5 − week 0	Adjusted *P* value	Difference: week 5 − week 0	Adjusted *P* value
UESCR threshold volume, mL	0.6 ± 0.1	**0.001**	0.5 ± 0.2	0.13
UESCR response latency, s	0.4 ± 0.1	0.052	0.4 ± 0.2	0.15
Peristaltic threshold volume, mL	0.4 ± 0.1	**0.001**	0.1 ± 0.1	0.99
Peristaltic response latency, s	0.3 ± 0.1	0.13	0.6 ± 0.1	**0.003**
LESRR threshold volume, mL	0.5 ± 0.1	**0.001**	0.2 ± 0.1	0.34
LESRR response latency, s	0.3 ± 0.2	0.31	0.6 ± 0.2	0.08
Respiratory response latency, s	0.2 ± 0.3	0.99	0.2 ± 0.3	0.99

Differences between baseline and follow-up data presented as LSMean ± SE using the repeated measures ANOVA model. *P* values represent Bonferroni adjustment with bold highlighting *P* < 0.05. Changes were noted at follow-up within both groups.

FM, feeding modification; LESRR, lower esophageal sphincter relaxation reflex; PPI, proton pump inhibitor; UESCR, upper esophageal sphincter contractile reflex.

**Table 6.**
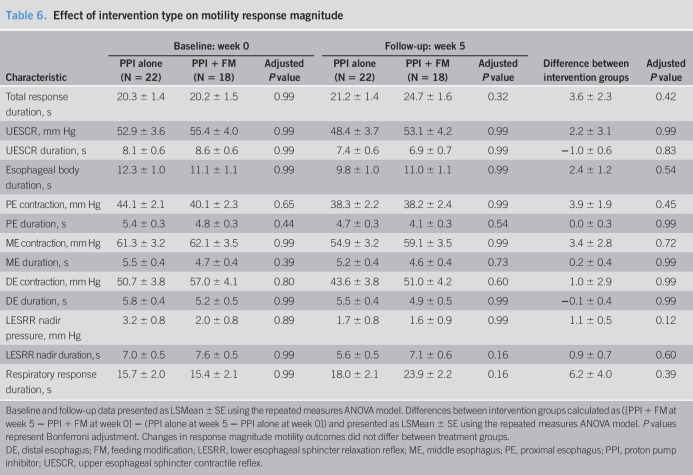
Effect of intervention type on motility response magnitude

Characteristic	Baseline: week 0			Follow-up: week 5			Difference between intervention groups	Adjusted *P* value
	PPI alone (N = 22)	PPI + FM (N = 18)	Adjusted *P* value	PPI alone (N = 22)	PPI + FM (N = 18)	Adjusted *P* value		
Total response duration, s	20.3 ± 1.4	20.2 ± 1.5	0.99	21.2 ± 1.4	24.7 ± 1.6	0.32	3.6 ± 2.3	0.42
UESCR, mm Hg	52.9 ± 3.6	55.4 ± 4.0	0.99	48.4 ± 3.7	53.1 ± 4.2	0.99	2.2 ± 3.1	0.99
UESCR duration, s	8.1 ± 0.6	8.6 ± 0.6	0.99	7.4 ± 0.6	6.9 ± 0.7	0.99	−1.0 ± 0.6	0.83
Esophageal body duration, s	12.3 ± 1.0	11.1 ± 1.1	0.99	9.8 ± 1.0	11.0 ± 1.1	0.99	2.4 ± 1.2	0.54
PE contraction, mm Hg	44.1 ± 2.1	40.1 ± 2.3	0.65	38.3 ± 2.2	38.2 ± 2.4	0.99	3.9 ± 1.9	0.45
PE duration, s	5.4 ± 0.3	4.8 ± 0.3	0.44	4.7 ± 0.3	4.1 ± 0.3	0.54	0.0 ± 0.3	0.99
ME contraction, mm Hg	61.3 ± 3.2	62.1 ± 3.5	0.99	54.9 ± 3.2	59.1 ± 3.5	0.99	3.4 ± 2.8	0.72
ME duration, s	5.5 ± 0.4	4.7 ± 0.4	0.39	5.2 ± 0.4	4.6 ± 0.4	0.73	0.2 ± 0.4	0.99
DE contraction, mm Hg	50.7 ± 3.8	57.0 ± 4.1	0.80	43.6 ± 3.8	51.0 ± 4.2	0.60	1.0 ± 2.9	0.99
DE duration, s	5.8 ± 0.4	5.2 ± 0.5	0.99	5.5 ± 0.4	4.9 ± 0.5	0.99	−0.1 ± 0.4	0.99
LESRR nadir pressure, mm Hg	3.2 ± 0.8	2.0 ± 0.8	0.89	1.7 ± 0.8	1.6 ± 0.9	0.99	1.1 ± 0.5	0.12
LESRR nadir duration, s	7.0 ± 0.5	7.6 ± 0.5	0.99	5.6 ± 0.5	7.1 ± 0.6	0.16	0.9 ± 0.7	0.60
Respiratory response duration, s	15.7 ± 2.0	15.4 ± 2.1	0.99	18.0 ± 2.1	23.9 ± 2.2	0.16	6.2 ± 4.0	0.39

Baseline and follow-up data presented as LSMean ± SE using the repeated measures ANOVA model. Differences between intervention groups calculated as ([PPI + FM at week 5 − PPI + FM at week 0] − (PPI alone at week 5 − PPI alone at week 0]) and presented as LSMean ± SE using the repeated measures ANOVA model. *P* values represent Bonferroni adjustment. Changes in response magnitude motility outcomes did not differ between treatment groups.

DE, distal esophagus; FM, feeding modification; LESRR, lower esophageal sphincter relaxation reflex; ME, middle esophagus; PE, proximal esophagus; PPI, proton pump inhibitor; UESCR, upper esophageal sphincter contractile reflex.

**Table 7.**
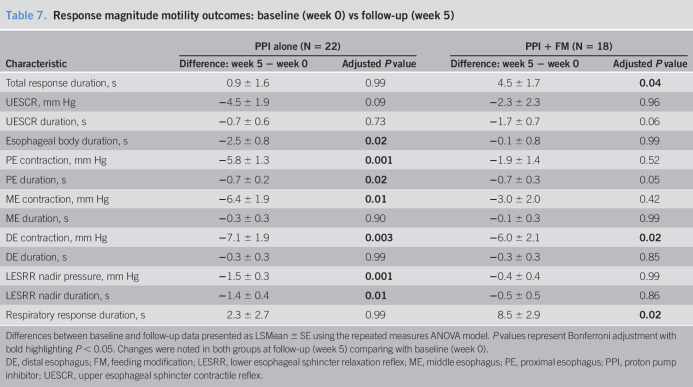
Response magnitude motility outcomes: baseline (week 0) vs follow-up (week 5)

Characteristic	PPI alone (N = 22)		PPI + FM (N = 18)	
	Difference: week 5 − week 0	Adjusted *P* value	Difference: week 5 − week 0	Adjusted *P* value
Total response duration, s	0.9 ± 1.6	0.99	4.5 ± 1.7	**0.04**
UESCR, mm Hg	−4.5 ± 1.9	0.09	−2.3 ± 2.3	0.96
UESCR duration, s	−0.7 ± 0.6	0.73	−1.7 ± 0.7	0.06
Esophageal body duration, s	−2.5 ± 0.8	**0.02**	−0.1 ± 0.8	0.99
PE contraction, mm Hg	−5.8 ± 1.3	**0.001**	−1.9 ± 1.4	0.52
PE duration, s	−0.7 ± 0.2	**0.02**	−0.7 ± 0.3	0.05
ME contraction, mm Hg	−6.4 ± 1.9	**0.01**	−3.0 ± 2.0	0.42
ME duration, s	−0.3 ± 0.3	0.90	−0.1 ± 0.3	0.99
DE contraction, mm Hg	−7.1 ± 1.9	**0.003**	−6.0 ± 2.1	**0.02**
DE duration, s	−0.3 ± 0.3	0.99	−0.3 ± 0.3	0.85
LESRR nadir pressure, mm Hg	−1.5 ± 0.3	**0.001**	−0.4 ± 0.4	0.99
LESRR nadir duration, s	−1.4 ± 0.4	**0.01**	−0.5 ± 0.5	0.86
Respiratory response duration, s	2.3 ± 2.7	0.99	8.5 ± 2.9	**0.02**

Differences between baseline and follow-up data presented as LSMean ± SE using the repeated measures ANOVA model. *P* values represent Bonferroni adjustment with bold highlighting *P* < 0.05. Changes were noted in both groups at follow-up (week 5) comparing with baseline (week 0).

DE, distal esophagus; FM, feeding modification; LESRR, lower esophageal sphincter relaxation reflex; ME, middle esophagus; PE, proximal esophagus; PPI, proton pump inhibitor; UESCR, upper esophageal sphincter contractile reflex.

**Table 8.**
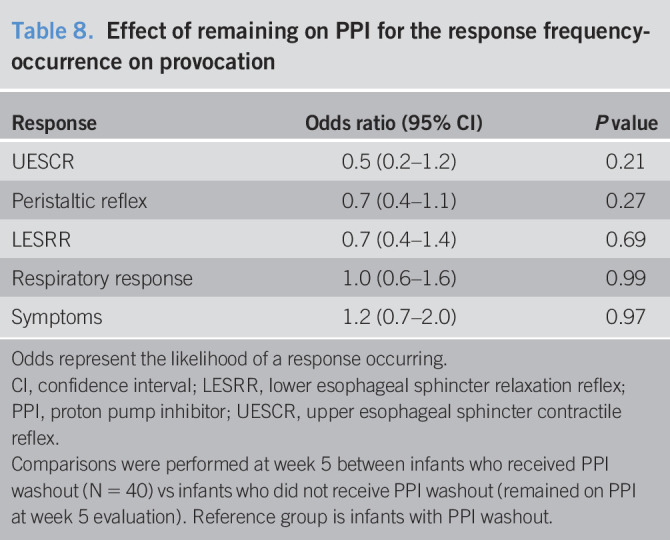
Effect of remaining on PPI for the response frequency-occurrence on provocation

Response	Odds ratio (95% CI)	*P* value
UESCR	0.5 (0.2–1.2)	0.21
Peristaltic reflex	0.7 (0.4–1.1)	0.27
LESRR	0.7 (0.4–1.4)	0.69
Respiratory response	1.0 (0.6–1.6)	0.99
Symptoms	1.2 (0.7–2.0)	0.97

Odds represent the likelihood of a response occurring.

CI, confidence interval; LESRR, lower esophageal sphincter relaxation reflex; PPI, proton pump inhibitor; UESCR, upper esophageal sphincter contractile reflex.

Comparisons were performed at week 5 between infants who received PPI washout (N = 40) vs infants who did not receive PPI washout (remained on PPI at week 5 evaluation). Reference group is infants with PPI washout.

Comparison of differences between and within intervention groups for response frequency-occurrence is shown (Tables 2 and 3): No significant differences were noted between intervention groups (Table 2). However, LESRR is less likely to occur at follow-up within both intervention groups (Table 3).

Comparison of differences between and within intervention groups for sensory metrics is shown (Tables 4 and 5): No significant differences were noted between intervention groups (Table 4); however, UESCR-, peristaltic-, and LESRR-threshold volumes increased in the PPI alone group, and peristaltic response latency increased in the PPI + FM group from baseline to week 5 (Table 5).

Comparison of differences between and within intervention groups for response magnitudes is shown (Tables 6 and 7): Response magnitudes were not different between groups (Table 6); however, distal esophageal contractile amplitudes were lesser at follow-up in both intervention groups (Table 7).

### Effect of PPI washout on select motility outcomes

Comparison of infants receiving PPI washout (N = 40) vs no washout (N = 9) for frequency-occurrence of UESCR, peristaltic reflex, LESRR, respiratory rhythm change, and symptoms is shown (Table 8). No significant differences in response occurrence for UESCR, peristaltic reflex, LESRR, respiratory response, or symptoms were noted.

## DISCUSSION

This randomized controlled trial was undertaken to examine the mechanistic effects of GERD therapies (conventional approach—acid suppression alone or study approach—acid suppression with FMs) on esophageal provocation–induced aerodigestive reflexes. We tested the hypothesis that midesophageal stimulation–induced aerodigestive reflexes (specifically esophageal peristaltic reflexes, UESCR, LESRR, respiratory changes, and symptoms) are superior in the study approach by comparing the differences (between week 5 and week 0) among intervention groups (conventional vs study) with the primary motility outcome as peristaltic reflex response. In addition, (i) to determine potential maturational effects, longitudinal comparisons (week 0 vs week 5) were performed within each therapeutic group; and (ii) to determine the effect of persistent PPI, comparisons were performed between infants remaining on PPI at week 5 (parental refusal to discontinue acid suppressive therapy) vs infants off PPI at week 5 (had PPI washout). Our hope was that specific therapies will then be possible in infants with objective GERD criteria. The salient findings and the pathophysiological basis of these comparisons are discussed below.

### Effects of GERD treatment bundle (acid suppression, feeding alterations, and postural changes) on mechanistic outcomes

Among infants with objectively defined GERD, the study approach (acid suppression + FMs) did not improve primary or secondary motility outcomes (aerodigestive reflexes—sphincteric, peristaltic, respiratory changes, or symptoms) to midesophageal provocations (simulated reflux). This is also consistent with our previous report of clinical outcomes (36), wherein symptom scores and feeding methods were not impacted.

### Pathophysiological basis for deterioration of sensory-motor aspects of aerodigestive reflexes in conventional and study groups

It has been shown before that the mechanisms of GER events in infants include hypotonic LES, transient LES relaxation (spontaneous relaxation >10 seconds), and swallow-associated LES relaxation (25,26). LES tonicity provides a distal barrier against refluxate entry into esophagus, while UES tonicity provides a proximal barrier against ascending refluxate (28,56). However, when esophagus is provoked through midesophageal stimulation of an abrupt bolus infusion (simulated GER event) or with GER events, a cascade of aerodigestive reflexes are triggered to facilitate safe bolus clearance; these reflexes include combinations of UESCR, peristalsis (EDR or SP), LES relaxation, and/or glottal closure manifesting as respiratory changes (31,54). As such, during these reflexes, symptoms may occur with functional or exaggerated responses and may serve as a protective mechanism. Albeit, when exaggerated protective mechanisms manifest with cardiorespiratory perturbations, those situations can be concerning for caretakers. However, we did not observe any adverse events in this study, and the presence of this self-regulatory mechanisms can be reassuring and is consistent with our recent observations on cardiorespiratory and pharyngoesophageal regulation mechanisms (46,57). Using the current study design, we were able to examine the occurrences, sensitivity, and magnitude of these reflexes in response to simulated GER through midesophageal infusions in those with objective evidence of GERD before and after conventional and study approaches.

From our previous physiological data using esophageal manometry methods in asymptomatic healthy neonates, UES tone averages 18–48 mm Hg (29), and LES tone averages 5–20 mm Hg and improves with maturation (33); the frequency-recruitment of UESCR, SP, and LESRR reflexes increased over a 4-week maturation by 36–38 weeks' PMA (30,33), while distal esophageal contraction amplitude remained unchanged over a 3- to 4-week period studied longitudinally (28). In addition, with maturation, the primary response to midesophageal-infusion transitions from primarily EDR responses to primarily SP responses, unlike adult studies where SP is the primary response. In the current study, given the diagnosis of GERD (objective determination), (i) the UES and LES sphincter lengths increased with maturation (Figure 3a) and the change in LES length was greater in the conventional group (Figure 3c); (ii) basal UES tone increased in conventional group but decreased in study group with maturation (Figure 3b) with change in UES tone more apparent in the conventional group (Figure 3d); and (iii) basal LES tone decreased in both groups (Figure 3b,d). Collectively, these findings support that GERD or its therapies may modify esophageal and sphincteric characteristics. This observation is also supported by the lower frequency-recruitment of LESRR (Table 3); frequency-recruitment of SP did not increase (Table 3) and worsened distal esophageal contraction amplitude (Table 7) at follow-up for both treatment groups. Use of placebo could have clarified if GERD or therapies made this contribution to esophageal pathophysiology.

It has been noted in older children and adults that chronic GERD has been associated with esophageal dysmotility and motor deficits (58–67), which could represent motility disorders. Furthermore, increasing GERD severity was associated with esophageal dysfunction and ineffective motility that improved with antacids or acid-suppressive therapy (59,67,68). This contrasts with our current study in which ARI severity and birth gestation were controlled for by the study-design allocation, and despite PPI therapy, infants have developed esophageal dysfunction and ineffective motility. PPI use converts acid-GER to non–acid-GER events, and esophageal provocation remains on treatment. Previous work in children and adults revealed that increasing GERD severity is associated with decreased LES tone (59,67) and decreases with age in GERD (69). In our study, the decreasing LES tone along with a decrease in distal esophageal amplitude in both groups point toward LES and distal esophageal as the most common pathophysiological targets in GERD. The decrease in frequency-recruitment of reflexes can be due to sensory or motor abnormalities or both (61).

### Effects of PPI washout vs continued use of PPI on the reflexes and symptoms

PPI washout was planned as per the trial design; however, about 18% parents refused discontinuation after 4 weeks. On examination of those infants who had PPI washout vs continued PPI at week 5 testing, there was no residual effect of washout on the reflexes. The study findings draw us to infer that the odds of UESCR, peristaltic reflexes, LESRR, respiratory responses, and symptoms remain the same despite being on PPI. Thus, the presence of reflexes is identical after washout, justifying that prolonged PPI therapy may not improve motility, symptoms, and respiratory changes.

GERD or PPI over 4 weeks may have modified the sensory thresholds despite washout. Refluxate can alter the mucosal permeability regardless of chemical nature and the distance between dilated intercellular spaces, as well as deeper permeation can alter the myenteric plexus functions (70–75). Our findings support that the conventional group indeed had greater sensory and motor dysfunctions in the esophagus, in that the esophageal amplitudes and duration of contractions were diminished at week 5 (Tables 6 and 7), thus supporting our hypothesis. Future studies must evaluate the role of mucosal stabilizers such as alginates or sucralfate compared with placebo (76,77). Mucosal protection may minimize esophageal dysmotility problems.

### Clinical and research implications

Restrictive feeding strategies and body positional modifications do not condition the esophagus or modify the esophageal provocation–induced reflexes aimed toward improving feeding and airway outcomes. In fact, the prevalence of supplemental oxygen and tube-feeding dependency was similar. Paradoxical changes in reflexes and their characteristics are seen with maturation despite therapy, and this may be attributed to the altered esophageal sensitivity thresholds or PPI use. Therefore, further randomized clinical trials in infants with objectively determined GERD (through pH-impedance metrics) and placebo are needed to support routine GERD management in this vulnerable population and prevent adverse effects because of altering gut microbiota (78) as maturation with proper nutrition may be a better alternative to improve sensory-motor motility aspects and decreasing GERD type symptoms. Although there are no safe prokinetics to promote esophageal motility, fundoplication is sometimes performed to improve LES barrier function and prevent refluxate in infants with poor airway protection, as in patients with chronic lung disease. However, the current study shows that those with GERD, distal esophageal peristalsis and LESRR become worse; therefore, fundoplication may exacerbate the problems because they are already having issues with downstream clearance.

We believe physiology/pathophysiology-guided approaches should be applied in managing GERD. Such approaches may specifically target improving nutrition and growth during maturation and or aimed to prevent esophageal-airway maladaptation mechanisms. Infants are a unique age group wherein nonverbal expression of aerodigestive and cardiorespiratory symptoms and signs pose greater challenges with diagnosis and empiric non–evidence-based therapies, for the lack of proper tools. Hence, the use of pharmacotherapies is greater in this group. There are clearly, potential differences in GERD etiology between infants, children, and adults, which may be related to varying maturational, maldevelopment, or maladaptive esophagogastric pathophysiology because of dietary, biologic, chemical, and environmental exposures during the life course. Indeed, studies in infants, older children, and adults are needed so as to ascertain the role of placebo in modifying the sensory-motor aspects of reflux that provokes aerodigestive defense mechanisms. Our study provides an investigational model that can be applied in older age settings, albeit with appropriate modifications.

### Strengths, limitations, and future directions

Data from this prospective study with a priori hypothesis and randomization of the therapy groups are a major strength. In addition, data analysis was performed by staff who were blinded to the group assignments. Multiple comparisons adjustments were made, and robust statistical methods applied to examine the hypothesis. Parental biases on returning for longitudinal studies or on discontinuing PPI therapy have resulted in lesser number of infants for longitudinal studies with or without PPI washout. However, we used the opportunity to examine infants remaining on PPI. We were not able to determine whether FMs only or maturation modify only responses because of the codependency of both on growth and functions. Future studies must include placebo in well-designed clinical trials in those infants tested objectively for GERD.

In conclusion, (i) acid suppressive therapy with FMs (volume restriction and positional changes) did not improve esophageal reflexes, respiratory changes, or symptoms. (ii) Despite acid suppressive therapy in both groups, distal esophageal and LES function worsened at week 5 at as evidenced by decreased distal esophageal contractility, lesser LES tone, and lesser frequency of LESRR. (iii) Continued PPI use does not modify frequency-occurrence of esophageal peristaltic, UES and LES reflexes, respiratory response changes, or symptom frequency on esophageal provocation. (iv) Further placebo trials are justifiable to determine the true effect of GERD pathophysiology and development of new treatment strategies.

## CONFLICTS OF INTEREST

**Guarantor of the article:** Sudarshan R. Jadcherla, MD, FRCPI, DCH, AGAF.

**Specific author contributions:** S.R.J.: developed proposal, obtained grant funding, conceptualized and designed the study, performed procedures and data acquisition, supervised data analysis, interpreted data, drafted the initial manuscript, edited and reviewed, and finally approved the final manuscript revision as submitted. K.A.H.: performed data acquisition, manometry data analysis and verification, interpretation of data, drafted the initial manuscript, and reviewed, and approved the final manuscript as submitted. I.K.G.: performed procedures and data acquisition, interpretation of data, edited and reviewed manuscript, and approved the final manuscript as submitted. R.H.: performed and verified manometry data analysis, verified data for statistical analysis, reviewed manuscript, and approved the final manuscript as submitted. H.I.: performed and verified manometry data analysis, verified data for statistical analysis, reviewed manuscript, and approved the final manuscript as submitted. V.Y.: performed independent statistical analysis, verification, interpretation, and manuscript writing, and approved the final manuscript as submitted. L.W.: supported with statistical study design, performed and guided with statistical analysis, reviewed and interpreted data, manuscript writing, and approved the final manuscript as submitted.

**Financial support:** Supported by the National Institutes of Health (R01 DK 068158 [to S.R.J.]) and the National Center for Advancing Translational Sciences (UL1TR002733 [to The Ohio State University Center for Clinical and Translational Science for REDCap support]).

**Potential competing interests:** None to report.

**Trial registry:** clinicaltrials.gov NCT02486263.

**Study Highlights**

**WHAT IS KNOWN**

✓ Diagnosis and management of gastroesophageal reflux disease (GERD) in intensive care unit infants is challenging and costly.
✓ Pharmacologic (acid suppression) and conservative (feeding modifications including restricted volumes, prolonged duration, and positional changes during prandial and postprandial states) management strategies are widely prevalent.
✓ The impact of acid suppression alone vs the bundle of acid suppression and feeding modifications on motility mechanisms, specifically the sensory-motor characteristics of esophageal reflexes (necessary for protection and clearance of GER events), is unknown.

**WHAT IS NEW HERE**

✓ In infants with GERD, acid suppressive therapy with feeding modification bundle does not alter esophageal reflexes vs acid suppressive therapy alone.
✓ Despite acid suppressive therapy in both groups, lower esophageal sphincter tone and distal esophageal contractility (common direct contiguous targets for reflux) worsened. Maturation is likely the key factor to strengthening defenses for GERD resolution in early infancy; further placebo studies are needed.
✓ Continued proton pump inhibitor use did not impact frequency-occurrence of responses or symptoms; prolonged use beyond 4 weeks is likely unwarranted.
